# Supplementary material for: Resistance to Radiation Enhances Metastasis by Altering RNA Metabolism
Source: bioRxiv. 2025 Feb 25:2025.02.19.638943. Preprint. [Version 1] doi: 10.1101/2025.02.19.638943 (PMC11888214; doi:10.1101/2025.02.19.638943)
Supplement: Supplement 1 [file NIHPP2025.02.19.638943v1-supplement-1.pdf]

bioRxiv preprint doi: https://doi.org/10.1101/2025.02.19.638943; this version posted February 25, 2025. The copyright holder for this preprint (which was not certified by peer review) is the author/funder, who has granted bioRxiv a license to display the preprint in perpetuity. It is made available under aCC-BY 4.0 International license.

Supplementary Figures

Supplementary Figure 1: Characterizing radioresistant and parental cell lines A. The diagram demonstrates the radiation dose escalation strategy that was implemented to develop the radioresistant cell lines. **B.** The percent of Annexin-V positive cells 48 hours after irradiation at a dose of 8Gy of the parental and radioresistant cell lines. n=3 independent samples. **C.** The PCA plots for the 4T1-RR, 4T1-parental, MDA-MB-468-RR, and MDA-MB0468-parental cell lines after differential gene expression analysis. **D.** The number of macro-metastatic sites observed in the thoracic cavity of the mice injected with either 4T1-RR or 4T1 cells. n=3 per section. **E.** The

bioRxiv preprint doi: https://doi.org/10.1101/2025.02.19.638943; this version posted February 25, 2025. The copyright holder for this preprint (which was not certified by peer review) is the author/funder, who has granted bioRxiv a license to display the preprint in perpetuity. It is made available under aCC-BY 4.0 International license.

bioluminescent images of the femur ex vivo from the mice injected with MDA-MB-468-RR vs MDA-MB-468.

Supplementary Figure 2: ITGβ3 expression and function in radioresistant TNBC A. An immunoblot of ITGβ3 and tubulin from the MDA-MB-468-RR cells and the MDA-MB-468 cells. **B.** An immunoblot of ITGβ3 and GAPDH from the 4T1-RR cells and the 4T1 cells. **C.** The expression of ITGβ3 after treatment with siRNAs (siCtrl, siITGB3-1, and siITGB3-2) and the migration capacity as determined by the scratch-wound assay (The scale bar is 200um). Data are represented as the mean ± SD. The P values in panel **C** were obtained by on-way ANOVA followed by Dunnett's multiple comparisons test.

Supplementary Figure 3: **ITGβ3 mRNA stability is reliant on HNRNPL expression A.** The enrichment plot for the geneset "REACTOME METABOLSIM OF RNA" that is upregulated in the MDA-MB-468-RR cells and 4T1-RR cells. **B.** The transcript stability of ITGβ3 as determined by the expression of ITGβ3 after different time points (0, 1, or 2 hours) of actinomycin d treatment in the radioresistant cells and parental cells based on RT-qPCR and normalized to expression at the 0 hour timepoint. **C.** Kaplan-Meier overall survival for breast cancer patients segregated by median HNRNPL expression in tumors taken from KM plotter. The correlation between ITGβ3 and HNRNPL expression in Basal Breast Cancer. **D.** The mRNA and protein expression of ITGβ3 when diminishing HNRNPL expression in the MDA-MB-468-RR cells **E.** The mRNA and protein expression of integrin β3 when diminishing HNRNPL expression in the 4T1-RR cells. **F.** An immunblot of HNRNPL expression in the MDA-MB-468 cells and the HNRNPL knockdown cells along with the flow cytometry data showing ITGB3 surface expression. **G.** An immunblot of HNRNPL expression in the 4T1T cells and the

bioRxiv preprint doi: https://doi.org/10.1101/2025.02.19.638943; this version posted February 25, 2025. The copyright holder for this preprint (which was not certified by peer review) is the author/funder, who has granted bioRxiv a license to display the preprint in perpetuity. It is made available under aCC-BY 4.0 International license.

HNRNPL knockdown cells along with the flow cytometry data showing integrin β3 surface expression. **H.** The mRNA expression of HNRNPL and ITGβ3 in the T47D cells transfected with empty vector of HNRNPL FLAG-tagged vector. **I.** The percent of cells expressing integrin β3 in the T47D cells transfected with empty vector of HNRNPL FLAG-tagged vector. Data are represented as mean ± SD. The P values in panel **B** were obtained from two-way ANOVA with Tukey's multiple comparisons tests, panel **D-G** were obtained from one-way ANOVA with Dunnett's multiple comparisons tests, panel **H** and **I** were obtained from student's t-test two-tailed.

Supplementary Figure 4: NRF2 binding at HNRNPL and lack of binding of HNRNPL to ITGβ3 transcript A. The enrichment plot for the geneset "REACTOME KEAP1 NRF2 PATHWAY" that is upregulated in the MDA-MB-468-RR cells and 4T1-RR cells. **B.** The correlation between NRF2 expression and HNRNPL expression in basal breast cancer patients taken from TIMER2.0. **C.** The ChIP-seq data from HeLa cells identifying the binding sites of NRF2 near the HNRNPL locus from GSE91997. The orange box designates the region where primers were designed to detect NRF2 binding near the HNRNPL promoter. **D.** The RIP-seq data from prostate cancer cells identifying the potential binding sites of HNRNPL near the ITGβ3 transcript from GSE72844. **E.** The RIP-qPCR data of potential HNRNPL binding sites near ITGβ3 taken from the MDA-MB-468-RR cells. Data are represented as mean ± SD. The P values in panel **E** were obtained unpaired t-test two-tailed.

Supplementary Figure 5: **The role of circRNAs in mediating ITGβ3 expression A.** The Pearson correlation of the circular RNA isoforms for each biological replicate of the HNRNPL knockdown cells and RR cells. **B.** The expression of the ceRNAs based on RT-qPCR in T47D

bioRxiv preprint doi: https://doi.org/10.1101/2025.02.19.638943; this version posted February 25, 2025. The copyright holder for this preprint (which was not certified by peer review) is the author/funder, who has granted bioRxiv a license to display the preprint in perpetuity. It is made available under aCC-BY 4.0 International license.

cells given empty vector or HNRNPL-FLAG plasmid. **C.** The expression of *circRAB12*, *circBACE2*, and *circBIRC6* in the control cells (siNC) compared to the siRNA knockdown cells (si-ceRNA-1 and si-ceRNA-2). **D.** The mRNA expression of *circRAB12* after transfection of the *circRAB12* or empty plasmid into the HNRNPL knockdown cells. The mRNA expression of ITGβ3 in those cells. Data are represented as mean ± SD. The P values in panel **B** and **D** were obtained unpaired t-test two-tailed, in panel **C** were obtained by one-way ANOVA with Dunnett's multiple comparisons test.

bioRxiv preprint doi: https://doi.org/10.1101/2025.02.19.638943; this version posted February 25, 2025. The copyright holder for this preprint (which was not certified by peer review) is the author/funder, who has granted bioRxiv a license to display the preprint in perpetuity. It is made available under aCC-BY 4.0 International license.

Supplementary Figure 1

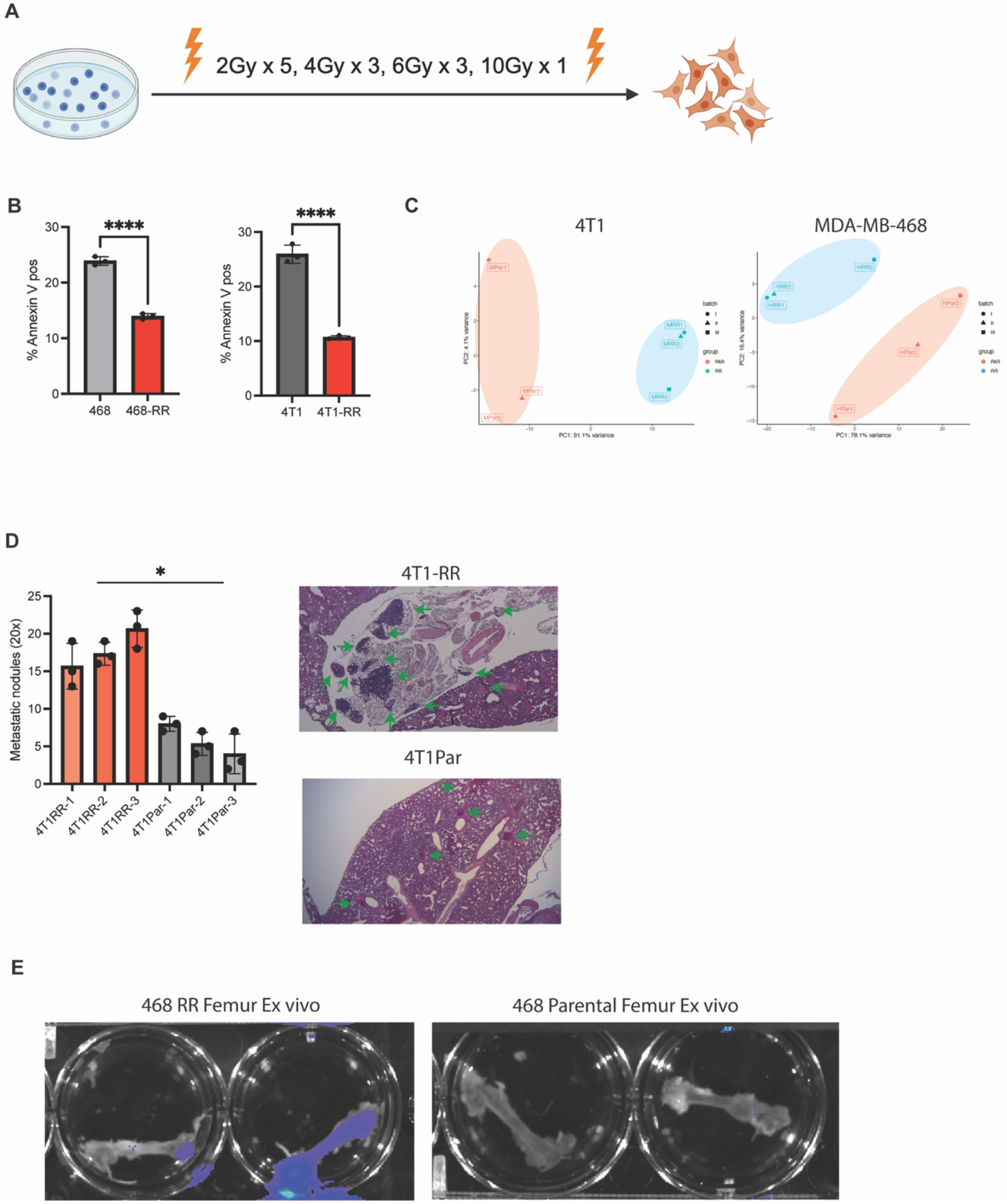

bioRxiv preprint doi: https://doi.org/10.1101/2025.02.19.638943; this version posted February 25, 2025. The copyright holder for this preprint (which was not certified by peer review) is the author/funder, who has granted bioRxiv a license to display the preprint in perpetuity. It is made available under aCC-BY 4.0 International license.

Supplementary Figure 2

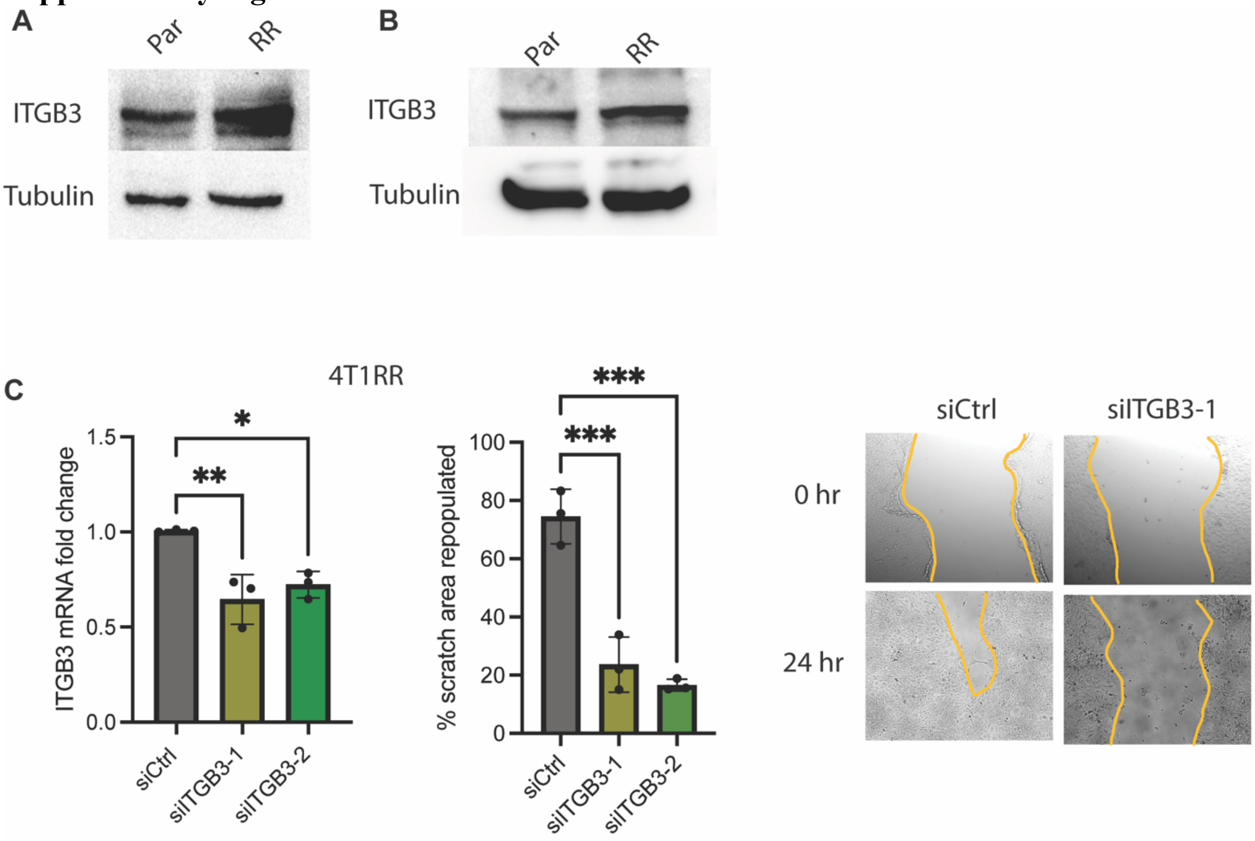

bioRxiv preprint doi: https://doi.org/10.1101/2025.02.19.638943; this version posted February 25, 2025. The copyright holder for this preprint (which was not certified by peer review) is the author/funder, who has granted bioRxiv a license to display the preprint in perpetuity. It is made available under aCC-BY 4.0 International license.

Supplementary Figure 3

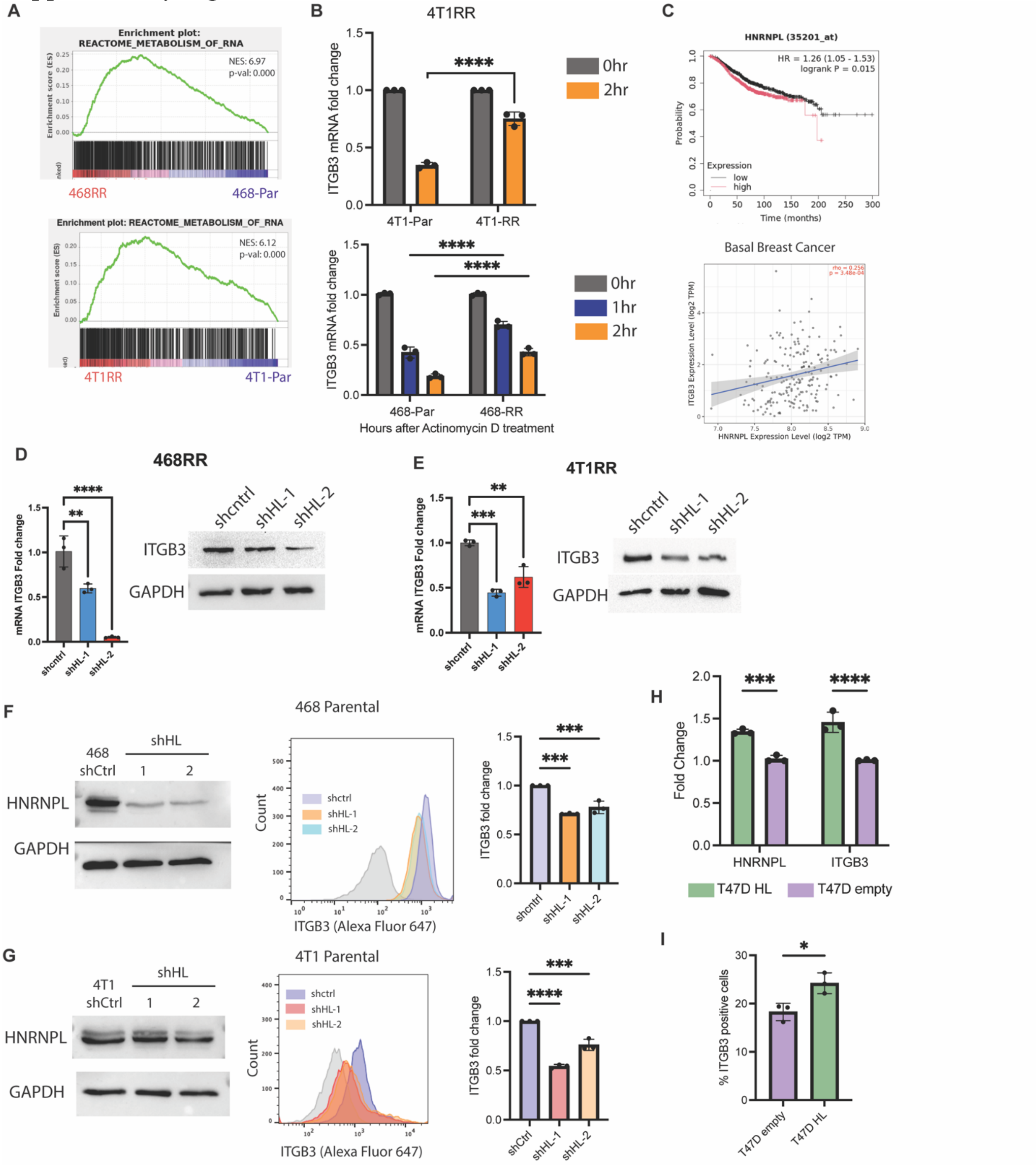

bioRxiv preprint doi: https://doi.org/10.1101/2025.02.19.638943; this version posted February 25, 2025. The copyright holder for this preprint (which was not certified by peer review) is the author/funder, who has granted bioRxiv a license to display the preprint in perpetuity. It is made available under aCC-BY 4.0 International license.

Supplementary Figure 4

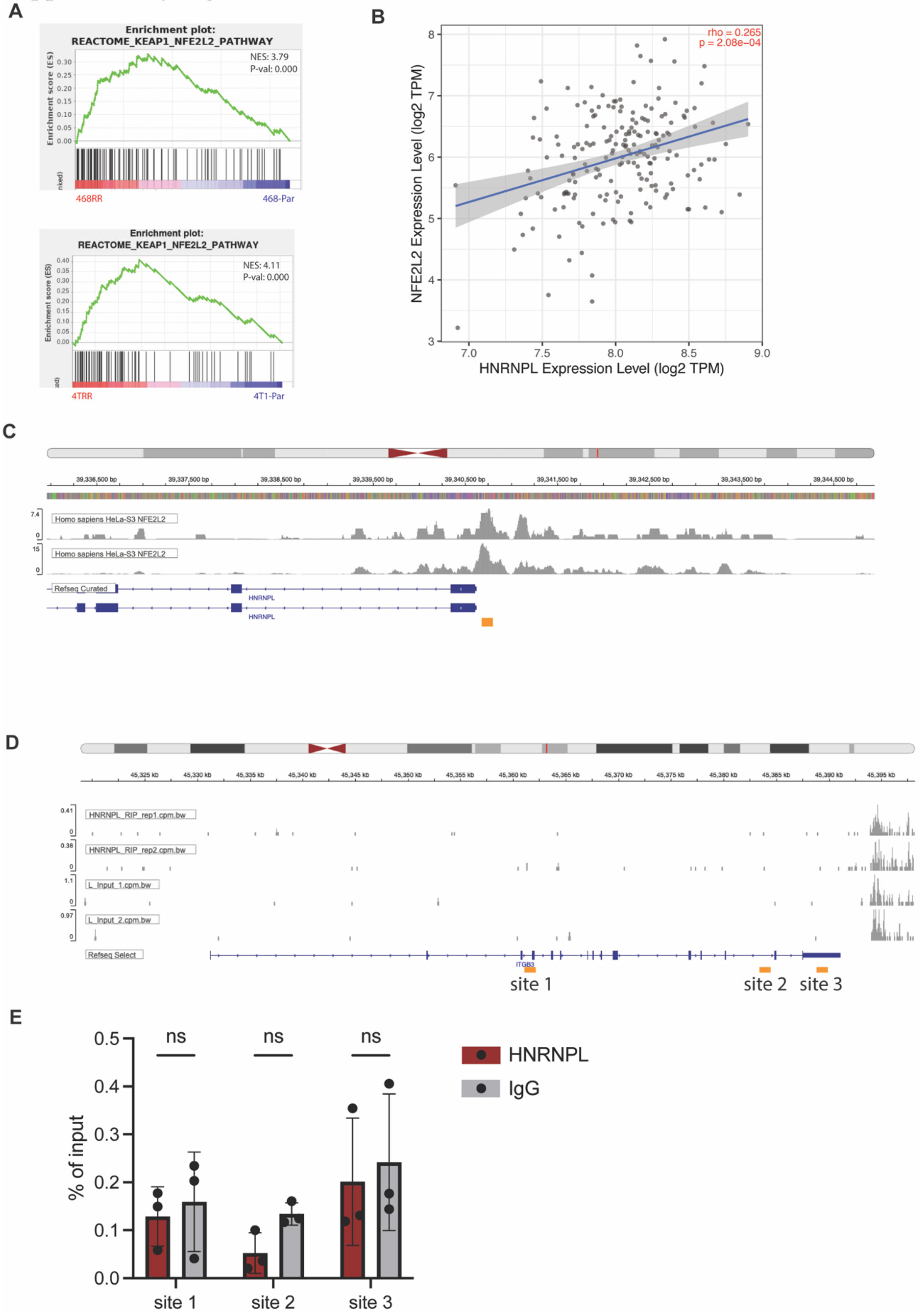

bioRxiv preprint doi: https://doi.org/10.1101/2025.02.19.638943; this version posted February 25, 2025. The copyright holder for this preprint (which was not certified by peer review) is the author/funder, who has granted bioRxiv a license to display the preprint in perpetuity. It is made available under aCC-BY 4.0 International license.

Supplementary Figure 5

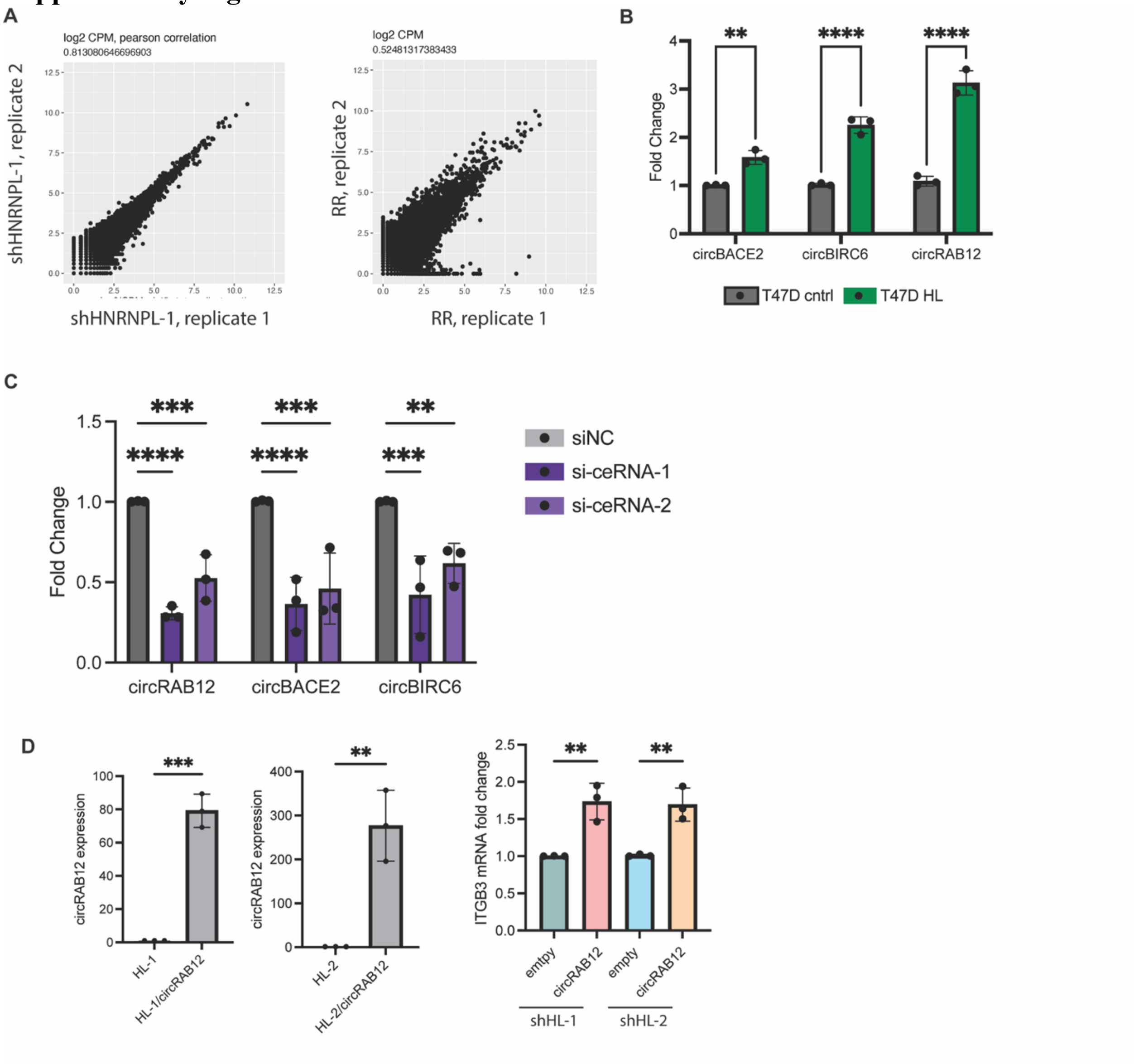

bioRxiv preprint doi: https://doi.org/10.1101/2025.02.19.638943; this version posted February 25, 2025. The copyright holder for this preprint (which was not certified by peer review) is the author/funder, who has granted bioRxiv a license to display the preprint in perpetuity. It is made available under aCC-BY 4.0 International license.

Supplementary Table 1: The scoring system for the capacity of circRNAs to sponge let-7 miRNAS

circ Name	number of sites	RR_exp	HL_exp	gene	score
chr18:8624938-8638416	6	103.597821	26.2057563	RAB12	464.352391
chr10:126970702-127061776	5	54.1132641	25.7065762	DOCK1	142.033439
chr1:108885275-108904254	5	44.1093492	19.4259052	GPSM2	123.41722
chr2:32377588-32395593	5	36.4562713	12.8836792	BIRC6	117.862961
chr22:20933779-20934244	5	38.9592926	16.1939178	CRKL	113.826874
chr16:81351583-81377702	4	43.6455674	17.9752679	GAN	102.681198
chr7:24623666-24680520	5	34.2716247	14.4807682	PALS2	98.9542826
chr21:41226266-41257326	4	32.4544117	10.2614285	BACE2	88.771933

Supplementary Table 2: The primers used for RT-qPCR, ChIP-qPCR, and RIP-qPCR.

Name	Forward primer	Reverse Primer
mITGB3	GGCGTTGTTGTTGGAGAGTC	CTTCAGGTTACATCGGGGTGA
mHNRNPL	TTGACGGAGTAGTGGAAGCTG	TCTTTGGCATCACCACCACAT
mNRF2	TCTTGGAGTAAGTCGAGAAGTGT	GTTGAAACTGAGCGAAAAAGGC
mGAPDH	AGGTCGGTGTGAACGGATTTG	TGTAGACCATGTAGTTGAGGTCA
ITGB3	AGTAACCTGCGGATTGGCTTC	GTCACCTGGTCAGTTAGCGT
HNRNPL	TACGCAGCCGACAACCAAATA	CTCCGGGAGTCATCCGAGT

bioRxiv preprint doi: https://doi.org/10.1101/2025.02.19.638943; this version posted February 25, 2025. The copyright holder for this preprint (which was not certified by peer review) is the author/funder, who has granted bioRxiv a license to display the preprint in perpetuity. It is made available under aCC-BY 4.0 International license.

NRF2	TCAGCGACGGAAAGAGTATGA	CCACTGGTTTCTGACTGGATGT
GAPDH	GGAGCGAGATCCCTCCAAAAT	GGCTGTTGTCATACTTCTCAT
circRAB12	TCCTTTCGCACTTTGTAATCCA	ATCTCTCCTGACCTGCTGTG
circBIRC6	ATCATCAGCTGCCTCATCTG	CCTTCCTACACCCTACTGCA
circBACE2	TGGAGGGCTTCTACGTCATC	TCTCTGTGTCAAAGTACGTGTC
HMOX1	AAGACTGCGTTCCTGCTCAAC	AAAGCCCTACAGCAACTGTCG
HL binding to ITGB3 site 1	AGAGCTTTGTGTTGAGGTGC	CCCTACCCTTCTCTGAATCCC
HL binding to ITGB3 site 2	AGGCTCAAACGATCCTCCC	GTTGCAGTGAACCGAGATCAs
HL binding to ITGB3 site 3	CGTCTCTCCTGATGTAGCACT	AGCTACTTATGGATTCCTCTTCC
NRF2 binding to HL	TAAGGAAATGGGCTGGAGGG	GGCCAGAGACAGTAAATGCAG

Supplementary Table 3: The siRNA sequences

siRNA	SEQUENCE
mITGB3-1	rCrUrArGrGrCrArArGrArArCrArUrUrArCrCrArArCrUrGAT
mITGB3-2	rArGrCrUrGrArCrGrGrArUrArCrUrGrGrCrArArArArArCGC

bioRxiv preprint doi: https://doi.org/10.1101/2025.02.19.638943; this version posted February 25, 2025. The copyright holder for this preprint (which was not certified by peer review) is the author/funder, who has granted bioRxiv a license to display the preprint in perpetuity. It is made available under aCC-BY 4.0 International license.

circRAB12-1	rArUrArUrArUrGrCrArArUrCrCrUrGrGrUrGrUrUrGrArCTT
circRAB12-2	rArUrCrCrUrGrGrUrGrUrUrGrArCrUrUrCrArArArArUrCAA
circBACE2-1	rGrArGrCrCrCrCrUrGrUrGrCrArGrUrArCrArGrArUrUrCTC
circBACE2-2	rCrGrArGrCrCrCrCrUrGrUrGrCrArGrUrArCrArGrArUrUCT
circBIRC62-1	rCrUrGrArUrGrArArCrCrUrUrGrUrArArArCrCrArGrGrUGG
circBIRC62-2	rUrGrArUrGrArArCrCrUrUrGrUrArArArCrCrArGrGrUrGGA
